# Incorporation of clinical features into a multivariate logistic regression model for the differential diagnosis of benign and malignant TI-RADS 4 thyroid nodules

**DOI:** 10.3389/fendo.2025.1550034

**Published:** 2025-05-29

**Authors:** Jun Hu, Xian Du, Yongbin Jiang, Yunle Wang, Lijuan Yang

**Affiliations:** Health Examination Center, Shanghai Health and Medical Center (Huadong Sanatorium), Wuxi, China

**Keywords:** TI-RADS 4 thyroid nodules, thyroid cancer, risk factors, clinical scoring model, diagnostic efficiency

## Abstract

**Objective:**

This study aimed to explore the diagnostic value of clinical features in the assessment of malignant thyroid Imaging Reporting and Data System (TIRADS) category 4 thyroid nodules and to provide a more effective reference for clinical diagnostic practices.

**Methods:**

A total of 998 patients with 1,103 TIRADS 4 thyroid nodules underwent conventional ultrasound (US) and clinical information assessment at the Shanghai Health and Medical Center from January 1, 2012, to June 30, 2024. A qualitative assessment of clinical and US features was performed, followed by univariable and multivariable logistic regression analyses using a training cohort, which contributed to the construction of the clinical TIRADS model. A receiver-operating characteristic (ROC) curve, a Hosmer-Lemeshow (HL) test and a decision curve analysis (DCA) were employed to further validate this model in the validation cohort.

**Results:**

Patient age, body mass index, sex, family history of thyroid carcinoma, and US features—such as vertical orientation, ill-defined or irregular margins or extrathyroidal extensions, microcalcifications, blood flow signals of central or peripheral vessels, and swollen cervical lymph nodes—were identified as independent risk factors in the clinical scoring model for TI-RADS 4 nodules. This diagnostic model achieved an area under the curve (AUC) of 0.943 [0.928, 0.959], with a sensitivity of 82.33%, specificity of 94.44%, diagnostic threshold of 5 points, accuracy of 87.42%, positive predictive value of 95.34%, and negative predictive value of 79.48% in the validation cohort. The HL tests and DCA also demonstrated excellent predictive performances.

**Conclusions:**

The integration of clinical and US features in the construction of the diagnostic model can significantly enhance the diagnosis of TIRADS 4 thyroid nodules and provide a reliable evaluation tool for clinical practice.

## Introduction

Due to the popularization of thyroid ultrasound examinations in health examinations, the detection rate of thyroid nodules was obviously increased in recent years (1, 2). Thyroid nodules are found in approximately 60% of the general population, with the malignancy rate of unselected thyroid nodule generally ranges from 1 to 5% (3–5),. The predominant pathological subtype of malignant cases is papillary thyroid carcinoma, which typically exhibits a low mortality rate, mild biological behavior, and is often asymptomatic throughout a lifetime (6, 7). Multiple studies (4, 7, 8) have indicated that a proportion of thyroid nodules are unnecessarily referred for surgery, resulting in an unfavorable risk and cost-benefit ratio. Given the high incidence, low invasiveness, and low mortality rates associated with thyroid nodules, there is a clear need for more cost-effective and risk-adapted management strategies for this prevalent condition.

Characterized by its non-invasiveness, convenience, repeatability, and low cost, ultrasound has become the first choice for screening thyroid nodules. To help diagnostic, ultrasound risk stratification guidelines have been developed in various countries worldwide (9–14). In 2020, the Society of Ultrasound in Medicine of the Chinese Medical Association published the Chinese guidelines for the Thyroid Imaging Reporting and Data System (C-TIRADS) (15). In C-TIRADS, thyroid nodules are categorized into classes 2 to 6 based on various ultrasound features. The nature of the class 2 and 6 nodules are definite, while class 3 nodules (malignancy rate is <2%) and class 5 nodules (malignancy rate is >90%) could also be diagnosed more readily through routine ultrasound. However, the ultrasound features of class 4 nodules exhibit some overlap, rendering the differentiation between benign and malignant nodules challenging.

Clinical factors (3, 16–20) such as patient age, sex, and family history, along with biochemical markers (17, 20) like TSH levels and thyroglobulin antibodies, have been found to be significant associated with malignancy risk of thyroid nodules. Prior research (19–21) has demonstrated that integrating these factors with ultrasound features can help clinicians make more informed decisions regarding diagnosis and management. Our research hopes to establish a model based on clinical and ultrasound risk factors to distinguish between benign and malignant class 4 thyroid nodules.

## Materials and methods

### Study design

This retrospective cohort study conducted at the Shanghai Health and Medical Center, China. We retrospectively analyzed the questionnaires and electronic medical records of patients with C-TIRADS 4 thyroid nodules who underwent regular health examinations at our hospital from January 1, 2012, to June 30, 2024, A total of 998 patients with 1,103 C-TIRADS 4 nodules were included for retrospective analysis.

The training cohort consisted of 447 thyroid nodule patients with pathologically confirmed malignancies from January 1, 2012, to December 12, 2020, were selected as the case group. In contrast, 324 thyroid nodule patients with pathologically confirmed benign confirmed benign nodules or those whose ultrasound characteristics remained unchanged for more than 9 to 10 years were selected as the control group. The validation cohort included 200 thyroid nodule patients with pathologically confirmed malignancies from January 1, 2021, to June 30, 2024, were selected as the case group. Meanwhile, 132 thyroid nodule patients with pathologically confirmed benign nodules were selected as the control group.

This study was approved by the Institutional Ethics Committee of the Shanghai Health and Medical Center, China. Our study conforms to the guidelines of the Declaration of Helsinki and its later amendments. Informed consent was obtained from all patients for being included in the study. The date of approval was 01 April 2022, reference number 2022(01).

### Participants

The research subjects were arranged by their respective companies to undergo annual health examinations at our hospital. All enrolled patients met the following inclusion criteria: (1) nodules classified as C-TIRADS 4, (2) age ≥ 18 years, and (3) availability of complete ultrasonography, clinical, and follow-up data. The exclusion criteria were set as follows: (1) a history of treatment with immunosuppressants, hormones, or iodine-containing medications, (2) a history of thyroid surgery or related radiotherapy, and (3) intolerance to puncture or surgical procedures.

### Participant data

Clinical data were obtained from electronic medical records of the physical examination center in our hospital. This dataset includes basic information, physical examinations, disease history, family history, medication history, thyroid ultrasound, and pathology reports.

Covariates: according to World Health Organization standards, age of patient was categorized into 18-44, 45-59, and ≥60 years. Body mass index (BMI) was categorized into 18.5-24.9, 25.0-29.9, and ≥30.0 kg/m^2^.

Ultrasound examinations were conducted by one thyroid ultrasound examiner and subsequently reviewed by another examiner with over ten years of experience. We implemented a standardized ultrasound reporting protocol based on the TIRADS guidelines to ensure diagnostic consistency (**Supplementary File Table S1**). Thyroid ultrasound image data, including nodule position, size, aspect ratio, composition, echogenicity, margins, calcifications, blood flow signals, and cervical lymph nodes, were meticulously recorded. Any differences between the two observers were re-evaluated collaboratively, discussed, and resolved through consensus. In cases where agreement could not be reached, a third, more experienced expert ultrasonographer made the final determination.

### Statistical analysis

Statistical evaluation was performed by SPSS 22.0. Variables exhibiting non-normal distribution were presented as median (interquartile range [IQR]), while normally distributed variables were shown as mean ± SD. Comparisons were made using independent samples. The *t*-test or Mann-Whitney *U* test was used for comparing continuous variables between the case and control groups. Categorical variables were analyzed using the *χ2* and Fisher’s exact tests. A *p* value <0.05 was considered statistically significant.

Additionally, within the training cohort, univariate logistic regression analysis was used to identify risk factors associated with malignant thyroid nodules. Factors with odds ratios>2 or P<0.1 were included in the multivariate regression analysis. Factors with *p <*0.05 in the multivariate analysis were considered as evaluation indices in the malignant thyroid nodules model. The regression coefficient (*β* value) for each factor was rounded to the nearest integer to establish the risk score for malignant thyroid nodules.

In the alidation cohort, the area under the curve (AUC) of the receiver operator characteristics (ROC) curve was used to assess the discriminative ability of the model, and the sensitivity, specificity, accuracy, positive predictive value, and negative predictive value of the model were calculated. A Hosmer-Lemeshow (H-L) test was used to evaluate the model’s calibration ability by assessing the closeness between the predicted prevalence rate and the observed prevalence rate. A decision curve analysis (DCA) was also used to evaluate the clinical utility of the model.

## Results

### Baseline demographics of study participants in the training cohort and validation cohorts

Our initial sample size included 1157 TIRADS 4 nodules, Of these, 16 nodules were excluded due to a prior history of treatment with iodine-containing medications (n =1), hormones (n =2) or thyroid surgery (n =13). Besides, 38 nodules were removed because of incomplete ultrasonography, clinical, or follow up data. In total, 447 malignant thyroid nodules and 324 control nodules were included in the training cohort. Additionally, 332 thyroid nodules were selected for the validation cohort, consisting of 200 malignant thyroid nodules and 132 control nodules.

**Table 1** presents the descriptive statistics for both the case and control groups. In the training and validation cohorts, no statistically significant differences were observed in BMI, waist circumference, hip circumference, height, smoking status, alcohol consumption, blood pressure, diabetes and thyroid function between the malignant nodule group and the negative control group. However, patients in the malignant nodule group were younger than those in the control group, with an average age of 46 years compared to 53.31 years in the training cohort (P <0.001), and 42.95 years compared to 52.29 years in the validation cohort (P <0.001). Furthermore, the proportion of females in the malignant nodule group was higher than that in the control group, both in the training cohort (49.7% vs. 40.1%, P =0.009) and in the validation cohort (46.0% vs. 34.8%, P =0.044).

**Table 1:** Clinical characteristics among the basic population.

Characteristics	Training cohort			Validation cohort		
	Normal control (n=324)	Malignant (n=447)	p value	Normal control (n=132)	Malignant (n=200)	p value
Age, mean ± SD, years	53.31 ± 10.13	46 ± 10.2	<0.001	52.29 ± 11.3	42.95 ± 10.65	<0.001
BMI, mean ± SD, kg/m2	24.5 ± 3.19	24.91 ± 3.8	0.119	24.76 ± 2.9	25.27 ± 3.86	0.193
Waistline, mean ± SD, cm	83.43 ± 9.18	82.8 ± 10.24	0.375	84.39 ± 8.62	83.41 ± 10.57	0.382
Hipline, mean ± SD, cm	95.74 ± 5.35	96.27 ± 5.61	0.307	96.75 ± 5.55	97.79 ± 6.27	0.383
Height, mean ± SD, cm	166.64 ± 8.76	165.86 ± 7.94	0.323	167.5 ± 8.16	168 ± 9.02	0.774
Systolic pressure, mean ± SD, mmHg	120.07 ± 15.08	118.15 ± 14.23	0.100	120.36 ± 15.39	118.88 ± 20.01	0.597
Diastolic pressure, mean ± SD, mmHg	75.03 ± 9.85	74.65 ± 9.55	0.624	74.7 ± 9.32	75.02 ± 10.27	0.839
Female sex, n (%)	130 (40.1%)	222 (49.7%)	0.009	46 (34.8%)	92 (46.0%)	0.044
Smoking history, n (%)	74 (22.8%)	116 (26.0%)	0.322	41 (31.1%)	43 (21.5%)	0.050
Drinking history, n (%)	136 (42.0%)	179 (40.0%)	0.601	57 (43.2%)	83 (41.5%)	0.761
Obesity (BMI≥30kg/m^2^), n (%)	15 (4.6%)	36 (8.1%)	0.059	6 (4.5%)	20 (10.0%)	0.070
Diabetes mellitus, n (%)	61 (18.8%)	73 (17.7%)	0.699	24 (18.5%)	22 (11.3%)	0.072
TSH, median (interquartile range), mIU/L	1.86 (1.25, 2.45)	1.76 (0.93, 2.47)	0.558	1.76 (1.12, 2.36)	1.93 (1.36, 2.75)	0.669
FT3, median (interquartile range), pmol/L	4.59 (4.24, 4.93)	4.54 (4.28, 4.97)	0.905	4.53 (4.35, 4.85)	4.71 (4.38, 5.28)	0.098
FT4, median (interquartile range), pmol/L	11.55 (10.48, 12.60)	11.51 (9.77, 13.33)	0.589	11.71 (10.35, 12.62)	11.18 (10.12, 11.88)	0.143
T3, median (interquartile range), nmol/L	1.55 (1.41, 1.72)	1.54 (1.40, 1.71)	0.247	1.59 (1.43, 1.71)	1.70 (1.51, 1.94)	0.083
T4, median (interquartile range), nmol/L	105.91 (94.05, 116.93)	109.28 (97.01, 120.93)	0.192	108.78 (97.74, 115.93)	102.49 (93.51, 113.33)	0.413

BMI, body mass index; TSH, thyroid stimulating hormone; FT3, free triiodothyronine; FT4, free thyroxin; T3, triiodothyronine, T4 thyroxin.

### Development of the clinical risk score model for TIRADS 4 nodules

#### Clinical and ultrasonographic risk factors for thyroid cancer explored by univariable logistic regression

In terms of clinical risk factors, age was found to be significantly and negatively correlated with the risk of malignant thyroid nodules. Additionally, being female was associated with an increased risk of malignant thyroid nodules, with an odds ratio (OR) of 1.472 (95% CI: 1.102, 1.967). Although obesity may be related to the presence of malignant nodules, it did not reach statistical significance. Concerning ultrasonographic risk factors, the following were identified as risk factors for malignant thyroid nodules: vertical orientation, blood flow in or around the nodule, microcalcifications, solid composition, ill defined or irregular margins or extrathyroidal extensions, and swollen cervical lymph nodes (all P < 0.05) (**Table 2**).

**Table 2:** Univariate and multivariate analysis to identify factors associated with malignant thyroid nodule and a scoring system developed from β coefficient in training cohort.

Variables	Univariate analysis		Multivariate analysis		β coefficient	Risk Score
	Odds ratio (95% CI)	p value	Odds ratio (95% CI)	p value		
Gender						
male	Reference		Reference			
female	1.472 (1.102-1.967)	0.009*	1.725 (1.021-2.914)	0.042*	0.545	1
Obesity (kg/m^2^)						
18.5~24.9	Reference		Reference			
25.0~29.9	1.053 (0.779-1.423)	0.739	1.289 (0.753-2.207)	0.355	0.254	0
≥30	1.842 (0.979-3.463)	0.058	3.699 (1.372-9.973)	0.010*	1.308	1
Age (years)						
≥60	Reference		Reference			
45~59	2.927 (1.902-4.504)	<0.001*	3.524 (1.604-7.745)	0.002*	1.26	1
≤44	8.353 (5.134-13.591)	<0.001*	13.437 (5.826-30.989)	<0.001*	2.598	3
History of smoking	1.184 (0.847-1.655)	0.323				
History of drinking	0.923 (0.69-1.235)	0.590				
Family history of thyroid carcinoma	2.916 (0.324-26.216)	0.339	43.433 (2.71-696.098)	0.008*	3.771	3
Ultrasound features of thyroid nodules						
vertical orientation	55.768 (13.666-227.576)	<0.001*	52.66 (10.487-264.44)	<0.001*	3.964	4
illdefined or irregular margins, or extrathyroidal extensions	65.33 (30.179-141.422)	<0.001*	56.751 (20.398-157.894)	<0.001*	4.039	4
blood flow signals of central or peripheral vessels	67.467 (16.555-274.955)	<0.001*	37.04 (7.507-182.762)	<0.001*	3.612	4
microcalcifications	12.906 (8.243-20.205)	<0.001*	20.3 (10.39-39.663)	<0.001*	3.011	3
solid composition	4.962 (2.501-9.844)	<0.001*	2.067 (0.786-5.436)	0.141	0.726	
markedly hypoechoic	6.637 (0.837-52.648)	0.073	8.013 (0.705-91.105)	0.093	2.081	
enlarged lymph nodes	7.541 (1.75-32.495)	0.007*	12.468 (1.06-146.624)	0.045*	2.523	3

CI, confidence interval; *P<0.05.

#### Clinical and ultrasonographic risk factors for thyroid cancer explored by multifactorial logistic regression

As shown in **Table 2**, we established the first clinical risk score model for TIRADS 4 nodules in Chinese individuals. Multivariate logistic regression analysis revealed that obesity, youth (defined as being under 44 years old), a family history of thyroid carcinoma, vertical orientation, blood flow in or around the nodule, microcalcifications, ill-defined or irregular margins or extrathyroidal extensions, and swollen cervical lymph nodes were independent risk factors for malignant nodules (all *P* < 0.05). We rounded the *β* values of these independent risk factors to the nearest integer to calculate the risk scores and subsequently created a scoring table.

### Validation of the clinical risk score model for TIRADS 4 nodules

**Figure 1:**
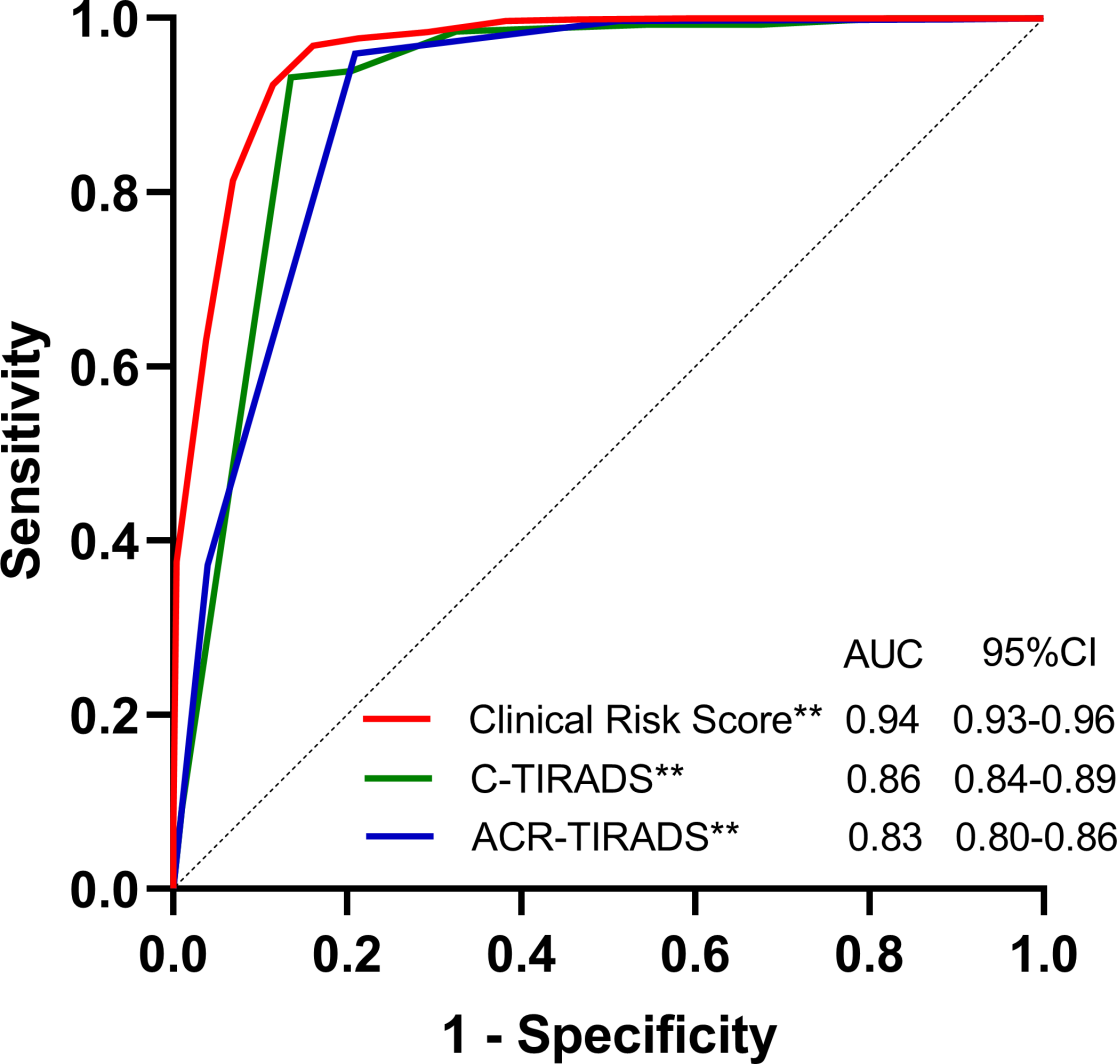
Area under the receiver-operating characteristic curve (AUC) of the Clinical Risk Score Model, C-TIRADS and the ACR-TIRADS in the validation cohorts. AUCs of the Clinical Risk Score Model, C-TIRADS and the ACR-TIRADS were 0.943(95% CI: 0.928, 0.959), 0.863(95%CI: 0.837, 0.890) and 0.833(95% CI: 0.804, 0.862), respectively. **P<0.001.

The final risk prediction models were evaluated within the validation cohorts to assess their validity (**Table 3**, **Figure 1**). In terms of discrimination, the risk score tested within the validation cohort achieved an AUC of 0.943 (95% CI: 0.928, 0.959). We estimated the optimal cut-off point, sensitivity, and specificity of the risk score model. At a cut-off point of 5, the sensitivity was 82.33% and the specificity was 94.44%. Additionally, the accuracy of the model was 87.42%, with a positive predictive value of 95.34% and a negative predictive value of 79.48%. The C-TIRADS and ACR-TIRADS classifications were also tested within the validation queues. The AUC of the C-TIRADS classification was found to be 0.863 (95% CI: 0.837, 0.890), with an optimal cut-off value of 2 points, a sensitivity of 76.96%, a specificity of 93.52%, an accuracy of 83.92%, a positive predictive value of 94.25%, and a negative predictive value of 74.63%. Moreover, the AUC of the ACR-TIRADS classification was 0.833 (95% CI: 0.804, 0.862), also with an optimal cut-off value of 2 points, a sensitivity of 72.93%, a specificity of 92.28%, an accuracy of 81.06%, a positive predictive value of 92.88%, and a negative predictive value of 71.19% (**Table 3**).

**Table 3:** Comparison of the diagnostic performances of TI-RADS in validation cohort.

Guidelinel (Cut off score)	Sensitivity	Specificity	Accuracy	PPV	NPV	AUC	p value
This clinical modelS (≥5 points)	82.33%	94.44%	87.42%	95.34%	79.48%	0.943(0.928-0.959)	<0.001
C-TIRADS (≥2 points)	76.96%	93.52%	83.92%	94.25%	74.63%	0.863(0.837-0.890)	<0.001
ACR-TIRADS (≥5 points)	72.93%	92.28%	81.06%	92.88%	71.19%	0.833(0.804-0.862)	<0.001

The data in parentheses represent a 95% CI value; PPV Positive predictive value, NPV Positive predictive value, AUC Area under the receiver-operating characteristic curve, C-TIRADS Chinese Society of Ultrasound in Medicine system, ACR-TIRADS the American College of Radiology Thyroid Imaging Reporting and Data System.

**Figure 2:**
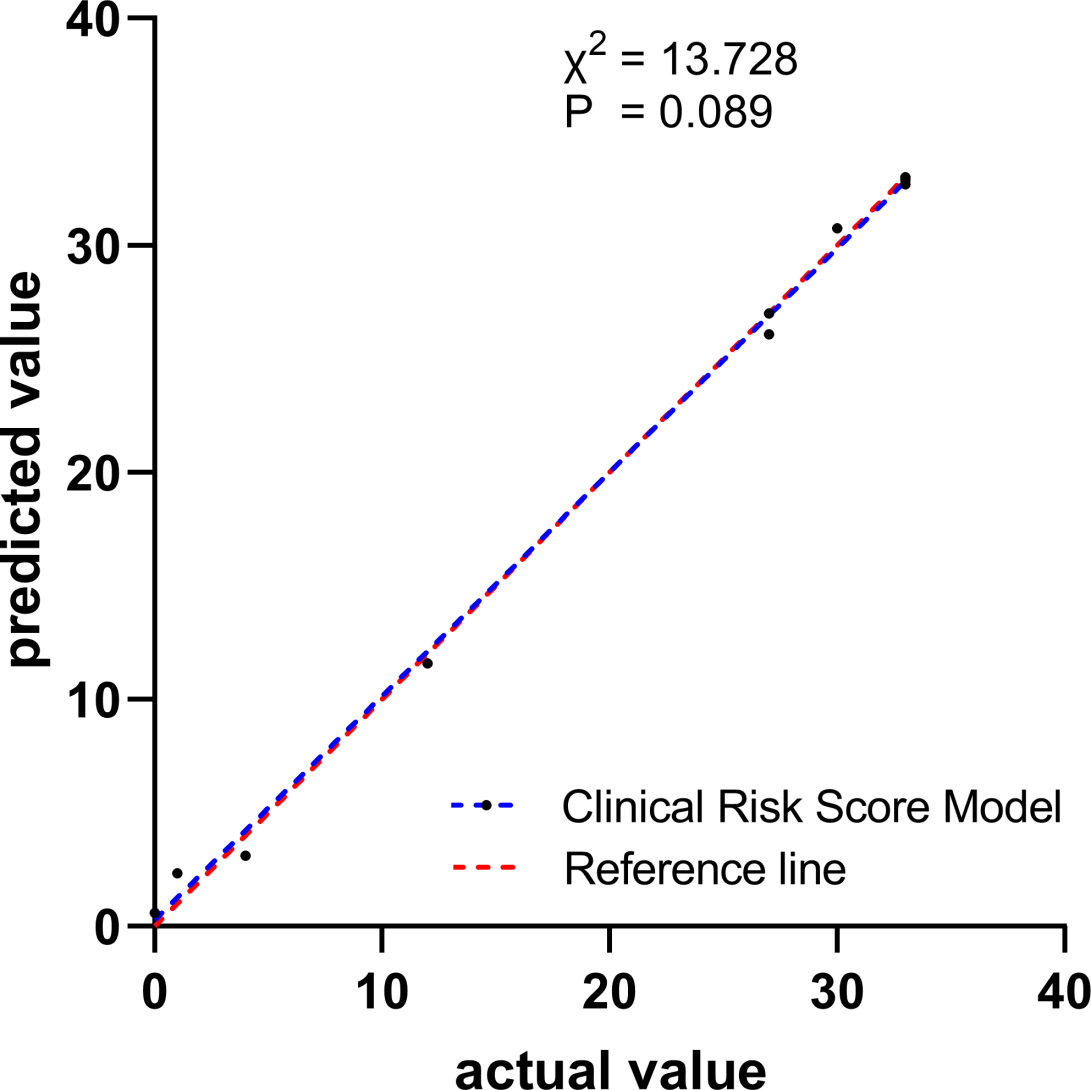
Calibration curve for observed versus predicted risk of developing thyroid cancer for Clinical Risk Score Model in the validation cohort.

**Figure 3:**
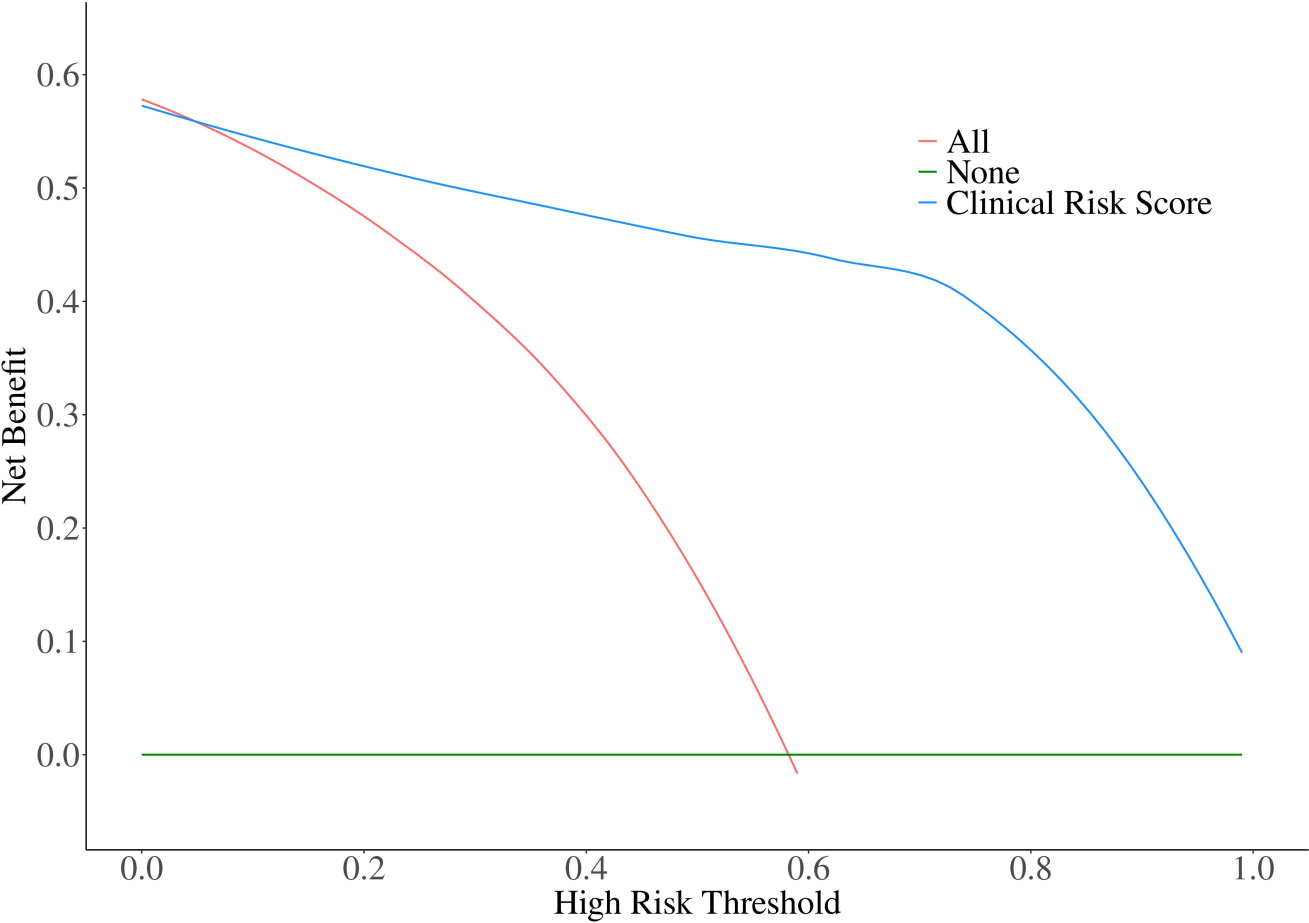
Decision curve analysis for Clinical Risk Score Model in the validation cohort. The net clinical benefit of patients was higher than that of the other two extreme curves in the vast majority of the threshold probability range.

In the validation cohort, the calibration of the risk score model was assessed using the H-L goodness-of-fit test, which indicated a good fit (*χ2 =* 13.728; p=0.089). We plotted a calibration graph using data from the validation cohort (**Figure 2**). In this calibration plot, the horizontal axis represents the actual observed values, while the vertical axis represents the model’s predicted values. The data points were randomly distributed around the reference line (y = x). The trend line drawn from these points closely overlapped with y = x, indicating that the actual values were in close proximity to the predicted values, demonstrating strong model calibration performance and external applicability. Furthermore, to evaluate the clinical applicability of the risk score model, we conducted a DCA in the validation set (**Figure 3**). We observed that the net clinical benefit of patients was higher than that of the other two extreme curves in the vast majority of the threshold probability range. These results validate that the clinical risk score model had a good clinical predictive ability, effectively balances diagnostic accuracy with reduced overtreatment burdens.

## Discussion

The risk score was a simple, non-invasive, and practical tool for evaluating thyroid nodules. In this study, we developed the first clinical and ultrasonographic risk score model to evaluate the nature of class 4 thyroid nodules in the Chinese population. The prediction model demonstrated both good discrimination and calibration capabilities for validation cohort.

### Clinical risk factors for thyroid cancer

Studies have found that low age (16, 17), female gender (3), radiation exposure (22), genetic predisposition (18), thyroid function and antibodies (17), and disease status (obesity, diabetes (23–28) are associated with an increasing risk of thyroid cancer. Radiation exposure was known as one of the most important risk factors for thyroid cancer, increasing the risk of malignant thyroid from 5% to 50% (22). In addition, thyroid function and antibodies may be associated with the risk of thyroid cancer. A recent study (18) found that thyroid stimulating hormone (TSH) ≥1.79 mIU/L (OR 1.76[1.05, 2.95]) and TgAb positivity (OR 2.59[1.25, 5.37]) were independent risk factors for malignant thyroid nodules. However, our study did not find a correlation between thyroid stimulating hormone and thyroid cancer. Obesity has been associated with the development of many cancers (23, 34), and insulin resistance, chronic systemic inflammatory states, and alterations in the gut microbiota may be the possible inner mechanisms (25). It has been estimated that for every 5% increase in BMI, the risk of thyroid cancer increases by 30% (26). Consistent with our study, a large US study (27) of 457,331 cases indicated that obesity was a significant contributor to the rapid increase in the incidence of papillary thyroid carcinoma between 1995-2015, both overweight and obese were significantly associated with papillary thyroid carcinoma (overweight OR 1.26 [1.05, 1.52]; obese OR 1.30 [1.05-1.62]). In our study, obesity did not present significance in the univariate analysis, but persist with multivariate analysis which indicating its inherent independent predictive value. A meta-analysis (28) of 10725884 cases from 16 studies showed that diabetes mellitus was associated with an increased risk of thyroid cancer (RR 1.20 [1.09, 1.33]), and especially more pronounced in women (RR 1.11 [1.06, 1.17]) than in men (RR 1.14 [1.00, 1.30]). The correlated molecular mechanism may be estrogen receptor alpha (ERα) and beta (ERβ), which have been reported to have an important role in the pathogenesis of thyroid cancer (29). Due to no significant differences were found between diabetes and thyroid cancer in our study, diabetes was not included in the new model.

### Ultrasound manifestations for thyroid cancer

A study conducted in 2021 found that ultrasound manifestations, including solidity, microcalcification, very hypoechoicity, blurred margins, irregular margins or extrathyroidal invasion, vertical position and other ultrasound features can indicate a high risk of malignant thyroid nodules (30). What’s more, angioinvasion is a critical indicator of aggressive behavior in differentiated thyroid cancer (DTC), correlating with distant metastasis and recurrence risks (31). Multiple studies have confirmed that blood flow signals (e.g., abnormal elevation of resistance index, the pulsatility index and peak systolic velocity) are associated with the invasiveness of thyroid cancer (32, 33). In our study, ultrasound features variables such as shape, margins, blood flow signals, calcifications, and cervical lymph nodes retained significance in both univariate and multivariate analysis, indicating their robustness as predictors. In contrast, while the composition variable was significant in univariate analysis, it failed to maintain significance in multivariate models, suggesting that its association may be confounded by other factors. In recent years, Chinese experts have proposed the C-TIRADS classification (15) to identify the nature of thyroid nodules recently, with a fluctuating ROC-AUC range of 0.753 to 0.933, a sensitivity of 84.25% to 93.1%, a specificity of 55.3% to 58.76%, an accuracy of 64.82% to 74.6% (34, 35). The C-TIRADS (15) guideline proposes that the probability of malignancy of thyroid nodules of category 4A, 4B and 4C are 2-10%, 10%-50%, and 50-90%, its diagnosis is still clinically difficult.

### Comparison and application of models

In recent years, machine learning models have been proved to be effective in thyroid nodule malignancy prediction. Current studies report AUC values ranging from 0.83 to 0.97 across different models (36–39), with accuracy rates between 71.7% to 92.1% in validation cohorts (38–40). However, machine learning models require substantial computational resources, and their performance depends on the image acquisition protocol and annotation quality. The clinical risk score model developed in our study achieves comparable performance, and is more feasible for clinical deployment. Besides, the incorporation of molecular markers, such as BRAF, RAS and TERT detection, enhances the diagnostic value of thyroid nodules exhibiting indeterminate cytology (41, 42), and plays a role in predicting outcomes for patients with papillary thyroid carcinoma (43). Nevertheless, the invasiveness, high cost, insufficient timeliness, and technical barriers associated with these methods limit their application as a first-line screening tool.

The addition of clinical features could enhance diagnostic efficiency for thyroid nodules (19–21). We applied the quantitative analysis parameters of ultrasonography and clinical features to construct a predictive risk score model for category 4 nodules. In the validation cohort, we found that the AUC, accuracy, sensitivity, specificity, positive predictive value, and negative predictive value of the new model were superior to those of the C-TIRADS and ACR-TIRADS stratification. In practice, sonographers perform initial evaluation based on predefined ultrasonographic features, while endocrinologists or surgeons could integrate clinical data to guide strategies on patient management and treatment. When the risk score of the new model reached 5 points, the Youden Index achieved its maximum value. Clinicians could prioritize patients with clinical risk score model scores≥5 points for biopsy or surgery. The diagnostic model could effectively avoid missed diagnoses when the score was 4 points and avoid misdiagnoses when the score was 6 points.

### Strengths and weaknesses

Our study has several strengths. The clinical risk score model exhibits good accuracy and calibration. In addition to ultrasound risk factors, the model also included age, gender, BMI and family history of thyroid carcinoma. Without increasing the burden of medical expenses, this study enhances the accuracy of the model in distinguishing between malignant and benign nodules compared to traditional C-TIRADS and ACR-TIRADS stratifications, thereby facilitating the development of more appropriate and beneficial treatment plans in daily clinical practice. Furthermore, the risk score model is highly practical. It serves as a simple and convenient assessment tool that can be easily understood and recognized by both doctors and patients in a short period, which may offer significant translational value for clinical practice.

Our study has several limitations. Firstly, as a single-center, retrospective study, it inevitably suffers from a lack of sample size and external validation, along with inherent biases such as incomplete data collection and recall bias. Secondly, the ultrasonic features were obtained from doctors with varying levels of experience in ultrasound imaging, which may introduce inter-observer variability. Thirdly, our risk score lacked indicators of ionizing radiation, as well as the morphology and blood flow characteristics of cervical lymph nodes. Finally, the pathological types of most malignant nodules were predominantly papillary thyroid carcinomas (92.5%), while other types of malignant thyroid tumors, such as follicular thyroid carcinoma (1.8%) and medullary thyroid carcinoma (1.2%), were not separately classified and analyzed due to the limited sample size. This limitation may restrict the generalizability of our model to other thyroid malignancies. Looking ahead, we hope to design prospective research, expand the sample size, include additional risk factors such as radiation history, the morphology and blood flow characteristics of cervical lymph nodes. In addition, future studies that incorporate surgical pathology and molecular markers may enhance the diagnostic and prognostic utility of the model.

## Data Availability

The raw data supporting the conclusions of this article will be made available by the authors, without undue reservation.
