# Supplementary material for: Incorporation of clinical features into a multivariate logistic regression model for the differential diagnosis of benign and malignant TI-RADS 4 thyroid nodules
Source: Front Endocrinol (Lausanne). 2025 May 29;16:1550034. doi: 10.3389/fendo.2025.1550034 (PMC12158692; doi:10.3389/fendo.2025.1550034)
Supplement: Supplementary file 1 [file DataSheet1.docx]

Supplementary Table S1

| Feature Category | Specific content | Operational Definition |
| --- | --- | --- |
| Shape | Wider-than-tall, Taller-than-wide | Anteroposterior/transverse ratio ≥1 in both transverse & longitudinal planes as "vertical orientation" |
| Margins | Smooth, Lobulated, Irregular, Extra-thyroidal | Discontinuity in ≥2 planes as "irregularity" |
| Blood flow signals | Absent, Peripheral, Central, Mixed | Semi-quantitative Adler classification: Grade 0 (none), 1-3 (peripheral to diffuse). Grade 1-3 as "positive blood flow signals of central or peripheral vessels" |
| Calcifications | None, Macrocalcifications, Peripheral, Punctate | Less than or equal to 1mm without posterior shadow as "microcalcifications" |
| Composition | Cystic, Spongiform, Mixed, Solid | Solid component ≥20% defined as "solid" |
| Echogenicity | Anechoic, Hyper- or Iso-, Hypo-, markedly hypoechoic | Quantitative comparison with strap muscles, echogenicity ≤the anterior cervical muscle group as "markedly hypoechoic" |
| Cervical lymph nodes | Normal, swollen | The short diameter of cervical lymph nodes ≥ 10mm as "enlarged lymph nodes" |


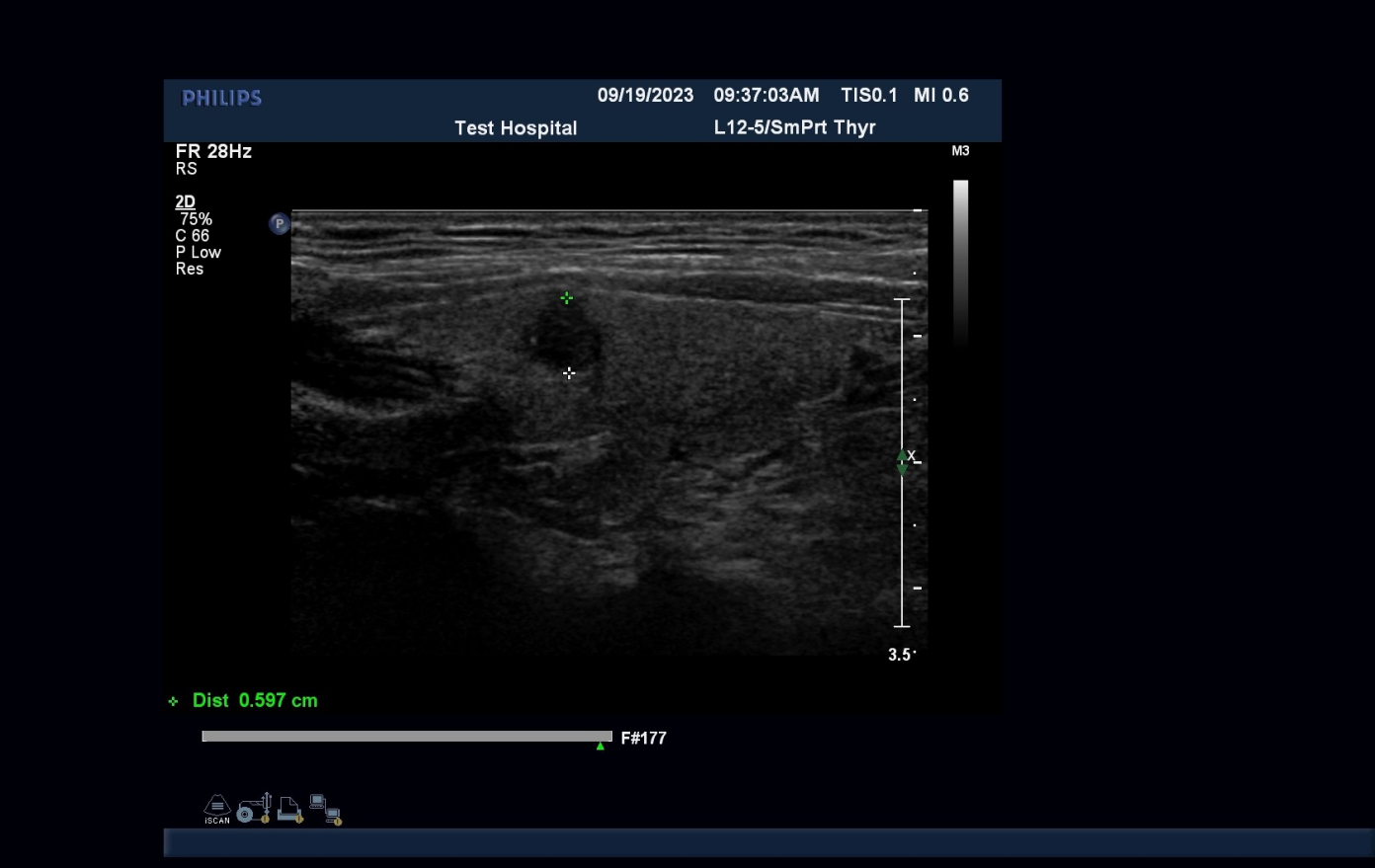

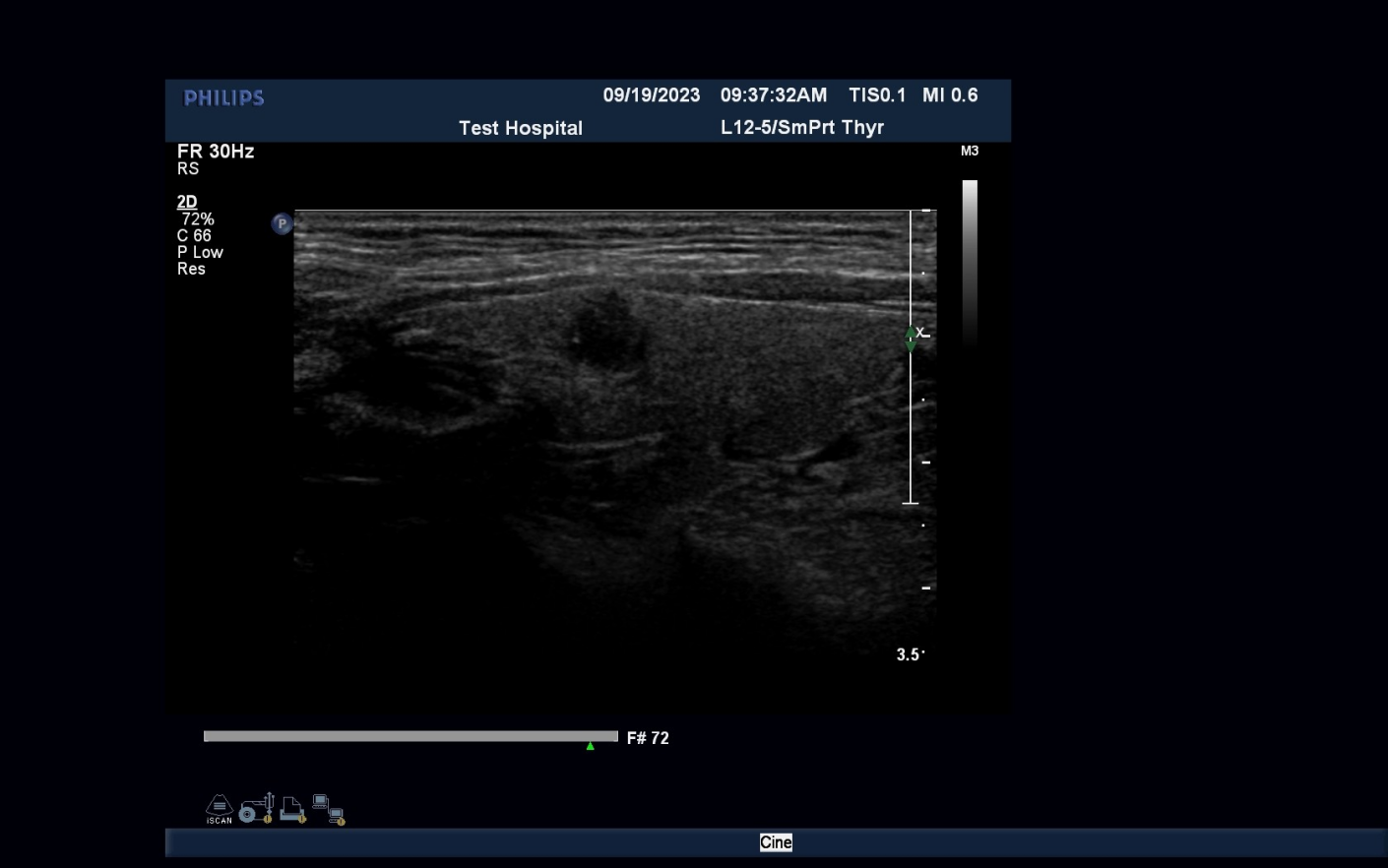

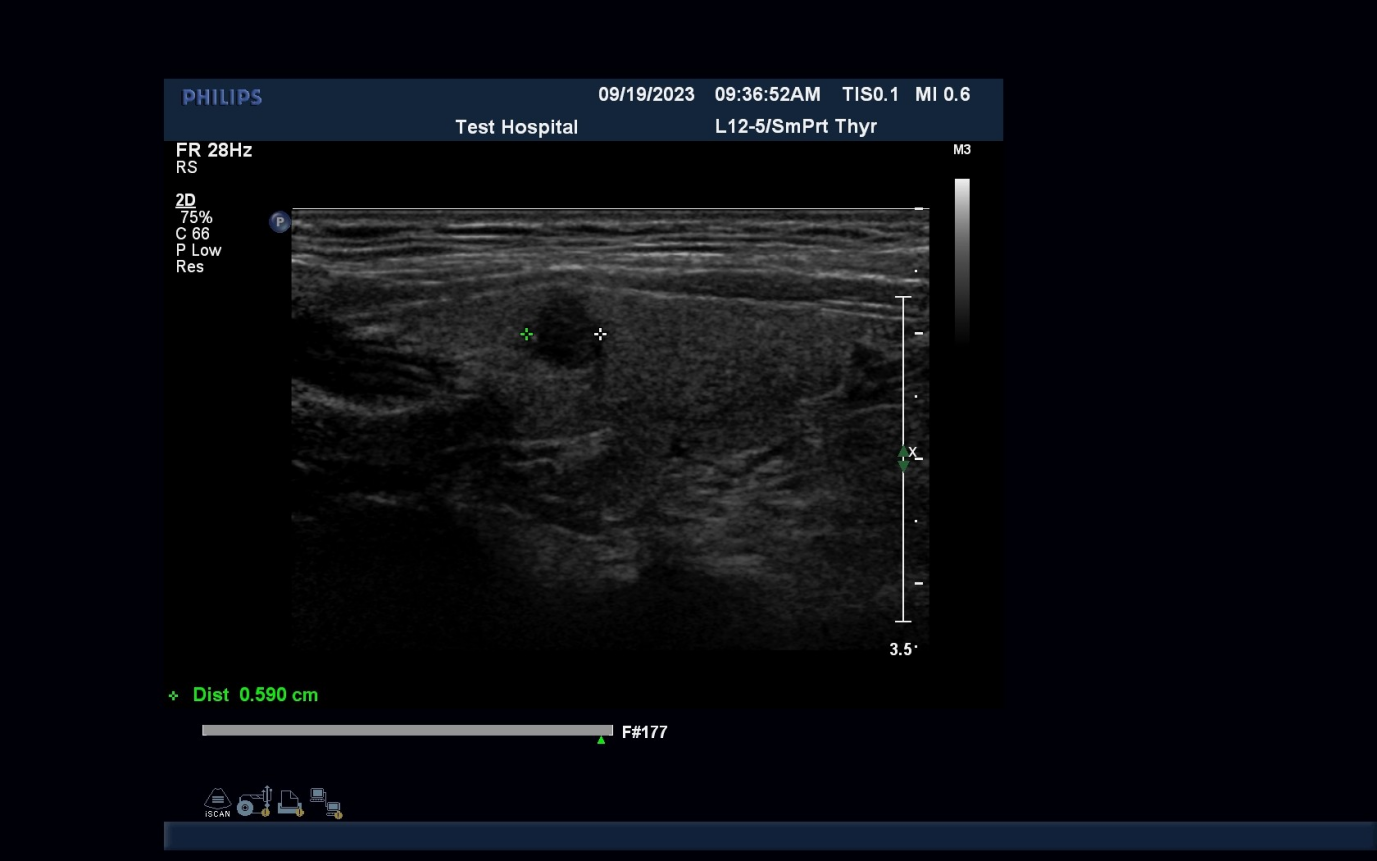


Supplementary Figure S1 A low echo can be seen in the right lobe of the thyroid gland, with a size of about 5.9 * 6.0mm, circular shape, full shape, uneven internal echo, clear boundary, smooth and complete, and no attenuation of the posterior echo. Color Doppler ultrasound shows no obvious abnormal color blood flow signals.No obvious plump lymph nodes were found around the thyroid gland and bilateral neck vessels. This nodule got a score of 1 in the clinical risk score model.


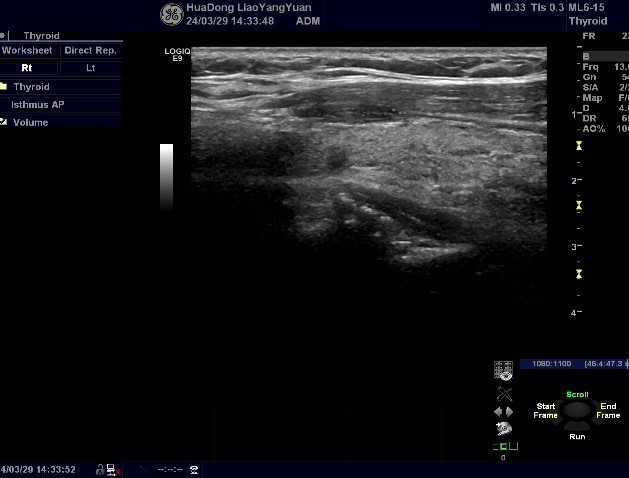

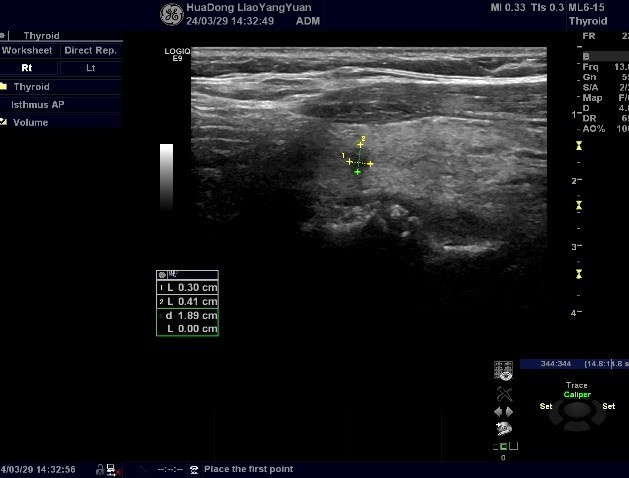


Supplementary Figure S2 A slightly low echo can be seen in the right lobe of the thyroid gland, with a size of about 3.0*4.1mm, an aspect ratio>1, an elliptical shape, uneven internal echo, unclear boundary, and no attenuation of the posterior echo. The remaining thyroid spots are thickened and unevenly distributed, and the vascular course is clear. Color Doppler ultrasound shows no obvious abnormal color blood flow signals. No obvious plump lymph nodes were found around the thyroid gland and bilateral neck vessels. This nodule got a score of 10 in the clinical risk score model.
